# A nomogram based on glycomic biomarkers in serum and clinicopathological characteristics for evaluating the risk of peritoneal metastasis in gastric cancer

**DOI:** 10.1186/s12014-020-09297-4

**Published:** 2020-09-19

**Authors:** Junjie Zhao, Ruihuan Qin, Hao Chen, Yupeng Yang, Wenjun Qin, Jing Han, Xuefei Wang, Shifang Ren, Yihong Sun, Jianxin Gu

**Affiliations:** 1 Department of General Surgery, Zhongshan Hospital, Fudan University, 180 Fenglin Road, Shanghai, 200032 China; 2 Key Laboratory of Glycoconjugate Research Ministry of Public Health, Department of Biochemistry and Molecular Biology, School of Basic Medical Sciences, Fudan University, 138 Yixueyuan Road, Shanghai, 200032 China; 3 Chinese Institute for Brain Research, Beijing, 102206 China

**Keywords:** Gastric cancer, Peritoneal metastasis, Glycomic analysis, MALDI-TOF–MS, Nomogram

## Abstract

**Background:**

Peritoneal metastasis (PM) in gastric cancer (GC) remains an untreatable disease, and is difficult to diagnose preoperatively. Here, we aim to establish a novel prediction model.

**Methods:**

The clinicopathologic characteristics of a cohort that included 86 non-metastatic GC patients and 43 PMGC patients from Zhongshan Hospital were retrospectively analysed to identify PM associated variables. Additionally, mass spectrometry and glycomic analysis were applied in the same cohort to find glycomic biomarkers in serum for the diagnosis of PM. A nomogram was established based on the associations between potential risk variables and PM.

**Results:**

Overexpression of 4 N-glycans (H6N5L1E1: m/z 2620.93; H5N5F1E2: m/z 2650.98; H6N5E2, m/z 2666.96; H6N5L1E2, m/z 2940.08); weight loss ≥ 5 kg; tumour size ≥ 3 cm; signet ring cell or mucinous adenocarcinoma histology type; poor differentiation; diffuse or mixed Lauren classification; increased CA19-9, CA125, and CA724 levels; decreased lymphocyte count, haemoglobin, albumin, and pre-albumin levels were identified to be associated with PM. A nomogram that integrated with five independent risk factors (weight loss ≥ 5 kg, CA19-9 ≥ 37 U/mL, CA125 ≥ 35 U/mL, lymphocyte count < 2.0 * 10 ~ 9/L, and H5N5F1E2 expression ≥ 0.0017) achieved a good performance for diagnosis (AUC: 0.892, 95% CI 0.829–0.954). When 160 was set as the cut-off threshold value, the proposed nomogram represented a perfectly discriminating power for both sensitivity (0.97) and specificity (0.88).

**Conclusions:**

The nomogram achieved an individualized assessment of the risk of PM in GC patients; thus, the nomogram could be used to assist clinical decision-making before surgery.

## Introduction

Although the incidence and mortality rate of gastric cancer have both decreased worldwide, gastric cancer remains the fifth most common malignancy and the third leading cause of cancer deaths in the world [1]. Peritoneal metastasis (PM) is the most frequent and lethal form of distant metastasis in patients with gastric cancer [2, 3]. Until recently, PM of gastric origin was regarded as an untreatable condition with a poor quality of life and short life expectancy [4]. PM was associated with a reported average overall survival time of approximately 4-5 months after diagnosis [3], which is far worse than the median survival of patients without distant metastasis.

Although computed tomography (CT), positron emission tomography-computed tomography (PET-CT), and magnetic resonance imaging (MRI) technologies have greatly improved, predicting peritoneal metastasis preoperatively remains difficult in clinical practice. A meta-analysis consisting of 15 CT studies and 4 PET-CT studies indicated that CT and PET-CT had a fairly low sensitivity for detecting peritoneal metastasis (0.33 and 0.28, respectively) [5]. Due to the low detection rate by routine examination methods, patients with peritoneal metastasis have to undergo exploratory operations, thus suffering from both mental and physical trauma as well as economic burden. Therefore, it is tremendously important to identify the presence of peritoneal metastasis preoperatively with non-invasive methods.

Recent research has preliminarily demonstrated the predictive effects of several clinical pathological characteristics, including tumour size, differentiation, histology type, and tumour biomarkers [2, 6], as well as inflammation-associated factors, such as neutrophil, lymphocyte, C-reactive protein, and albumin levels, as well as neutrophil to lymphocyte ratio (NLR) [7, 8]. However, a single or even a few selected markers cannot define all the characteristics of peritoneal metastasis. Thus, systematic analysis of peritoneal dissemination is essential.

Serological glycomic profiling is an emerging non-invasive screening tool for finding potential biomarkers in a variety of cancers [9–11]. Serum glycan alteration has been reported to play an important role in regulating tumour proliferation, invasion, and metastasis [9, 12]. In our previous work, we identified several glycan biomarkers by using a modified mass spectrometry method (matrix-assisted laser desorption/ionization time-of-flight mass spectrometry: MALDI-TOF MS) for the early detection and progression surveillance of gastric cancer [13].

In this study, we aimed to discover peritoneal metastasis associated glycan biomarkers by using glycomics analysis based on the MALDI-TOF MS method and clinicopathologic characteristics to establish a nomogram for predicting the risk of peritoneal metastasis in gastric cancer patients.

## Materials and methods

### Patient selection

**Fig. 1:**
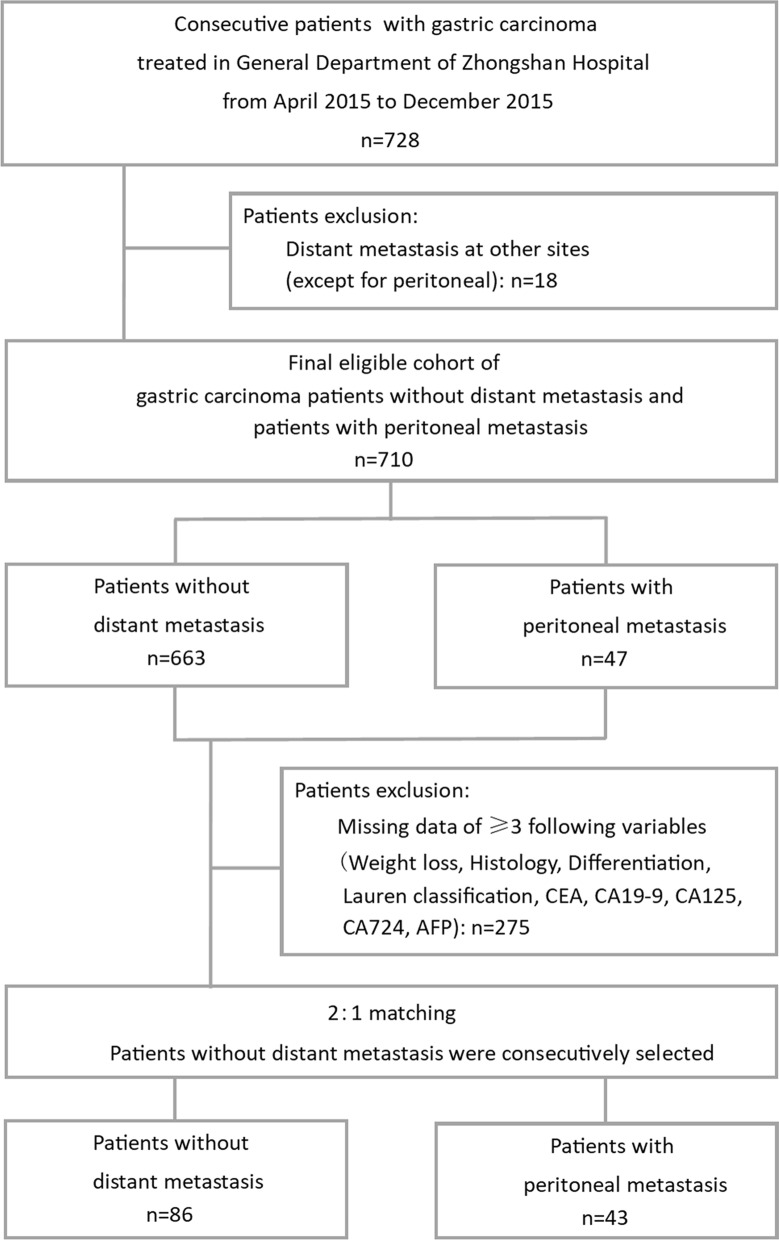
Diagram of study population and cohort selection, with exclusion criteria described on the right-hand side of the diagram. *AFP* alpha-fetoprotein, *CEA* carcinoembryonic antigen, *CA19*-*9*, *CA125*, *CA724* carbohydrate antigen 19-9, 125, and 724

The clinical and pathological data of a consecutive cohort of 728 gastric cancer patients who were initially treated in the general department of Zhongshan Hospital from April to November 2015 were retrospectively collected. Patients with distant metastasis at other sites (except for peritoneal metastasis) and with three or more missing clinical data were excluded. We then applied a 2:1 ratio of non-distant metastasis patients (n = 86) and peritoneal metastasis patients (n = 43) for further examination and analysis. Patients with peritoneal metastasis were consecutively selected. The exclusion criteria and study cohort flow diagram are summarized in Fig. 1. The use of human serum samples and clinical data was approved by the ethics committee of Zhongshan Hospital, Fudan University and was performed in accordance with the ethical standards presented in the 1964 Declaration of Helsinki and its later amendments. Informed written consent from all participants was acquired.

### Diagnosis of peritoneal metastasis

Every patient received a CT or PET-CT scan before surgery. When suspected peritoneal metastasis was reported in the examination, our multidisciplinary team (MDT) then discussed the case. If the evidence of PM was regarded as sufficient by the MDT, then the diagnosis was made; in contrast, if the evidence was not sufficient, diagnostic laparoscopy was used. When peritoneal metastasis was not reported in the examination, exploratory laparotomy or diagnostic laparoscopy accompanied by peritoneal lavage and exfoliative cytologic examination was applied before gastrectomy was performed.

### Mass spectrometry and Glycomic analysis

N-glycans were released from serum glycoproteins and derived by ethyl esterification reagent according to the method described in previous study [13]. Briefly, 10 μL serum was denatured in 30 μL denaturation buffer with 2% SDS, followed by digestion in 20 μL released mixture with 0.5 mU PNGase F (Roche Diagnostics, Mannheim, GER). The released glycan sample was added into tubes filled with 200 μL freshly prepared derivatization reagent (250 mM EDC and 250 mM HOBt in ethanol). Before MS analysis, the obtained oligosaccharides were purified by Sepharose HILIC SPE (45–165 μm, GE Healthcare, Uppsala, SE). Ethyl esterfied N-glycans were subjected to MS analysis by AXIMA Resonance MALDI-TOF MS (Shimadzu Corp, Kyoto, JPN) equipped with a 337 nm nitrogen laser in reflector positive ionization mode. Samples were analyzed in triplicate. The m/z range was monitored to span from 500 to 5000. The MALDI-TOF MS data were pre-processed, normalized and extracted using the software of Progenesis MALDI (Nonlinear Dynamics Ltd, Newcastle Upon Tyne, UK). The GlycoWorkbench software was used for the annotation of MS spectra (H = hexose, N = *N*-acetylhexosamine, F = fucose, L = α 2,3-sialic acid, E = α 2,6-sialic acid).

### Statistical analysis

Clinicopathologic characteristics between non-distant metastasis and peritoneal metastasis groups were compared by using Pearson’s χ^2^ test for categorical variables and Mann–Whitney test for continuous variables. A forest plot was created according to the result of multivariable logistic analysis. A nomogram was created with R software (R Studio, Boston, MA, USA) using the ‘rms’ package to be a new prediction model. A calibration plot was generated to examine the performance characteristics of the nomogram. Receiver operating characteristic (ROC) curve was used to compare the sensitivity and specificity for the prediction of PM by the parameters. Area under the curve (AUC) was used to judge the predictive accuracy of different parameters. A two-sided significance level of 0.05 was used in all statistical tests. Statistical analysis were performed using SPSS package (Version 22, SPSS Inc., Chicago, IL, USA) and R package.

## Results

### Patient characteristics and diagnostic value of CT or PET-CT

A total of 728 gastric cancer patients, which included 663 (91%) non-metastatic patients and 65 (9%) distant metastatic patients, were consecutively treated in the General Department of Zhongshan Hospital from April 2015 to December 2015. The TNM stage and distant metastasis details of the patients are depicted in Additional file 1: Table S1. Notably, peritoneal metastasis accounted for up to 72% (47/65) of all distant metastasis patients.

Among the 47 patients with peritoneal metastasis, 25 patients received a CT scan, 12 patients received a PET-CT scan, and 10 patients underwent both examinations preoperatively. Nine cases of PM were detected by CT scans, with a detection rate of 25.7% (9/35), and ten cases were detected by PET-CT scans, with a detection rate of 45.5% (10/22). Importantly, all metastatic cases detected by imageological examination were finally confirmed by surgery with 100% accuracy. The results indicated that CT or PET-CT had a low sensitivity but extraordinarily high accuracy for peritoneal metastasis diagnosis. Due to the low detection rate of the CT or PET-CT scan, more than 60% (29/47) of patients had to undergo invasion examinations. Among them, the majority of patients (28/29) were observed to have macroscopic lesions with peritoneal metastasis.

After 2:1 cohort matching, 86 non-metastatic patients consecutively treated from May to June and 43 patients with peritoneal metastasis were finally recruited for further analysis.

### Glycomics analysis of mass spectrometry related to peritoneal metastasis

The *N*-glycomic profiles were identified by MALDI-MS according to our previous study [13]. Based on the comprehensive glycomic analysis, we totally examined 81 N-glycans (peaks) in all serum samples. The typical glycomic profiles and the glycan structures and compositions were presented in Additional file 1: Figure S1 and Table S2.

**Fig. 2:**
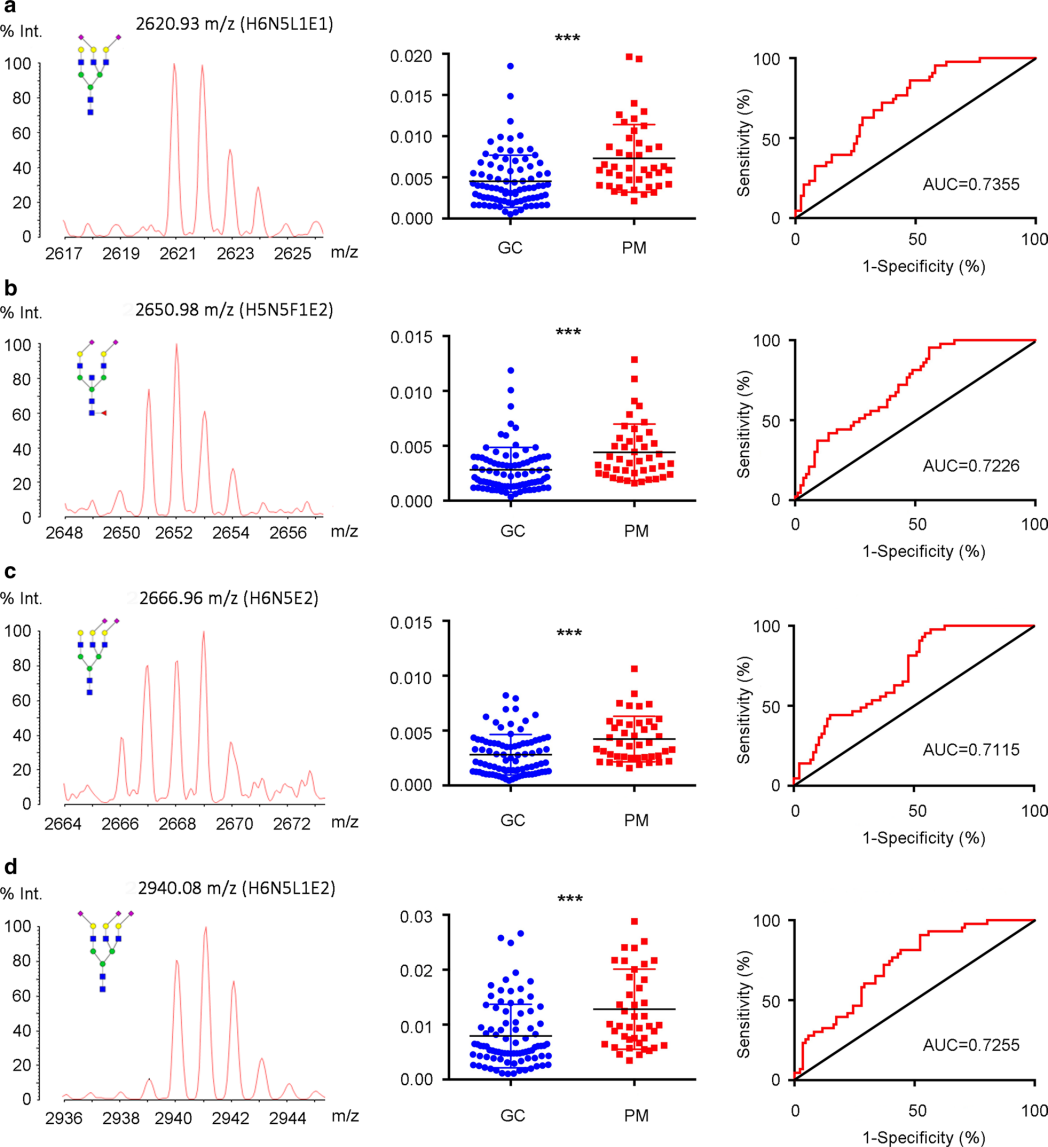
Representative mass spectra, scatter plot and ROC (Receiver Operating Characteristic) curve analysis of top four most prominently different expression of glycoforms between non-metastatic GC patients and GC patients with peritoneal metastasis. **a** 2620.93 m/z (H6N5L1E1). **b** 2650.98 m/z (H5N5F1E2). **c** 2666.96 m/z (H6N5E2). **d** 2940.08 m/z (H6N5L1E2) were identified. *GC* gastric cancer, *PM* peritoneal metastasis, *AUC* area under ROC curve, *H* hexose, *N* *N*-acetylhexosamine, *F* fucose, *L* α 2,3-sialic acid, *E* α 2,6-sialic acid. ***P < 0.001

The results of the individual N-glycan structure abundance analysis showed that 22 types of glycans displayed significant differences between non-metastatic and peritoneal metastasis gastric cancer (*P* < 0.05) (Additional file 1: Table S3). The *N*-glycans demonstrating both *P* < 0.0001 and AUC > 0.7 (H6N5L1E1: m/z 2620.93; H5N5F1E2: m/z 2650.98; H6N5E2, m/z 2666.96; H6N5L1E2, m/z 2940.08) were considered valuable for predicting peritoneal metastasis and were included for further analysis (Fig. 2a–d).

### Univariable and multivariable logistic analysis for peritoneal metastasis

**Fig. 3:**
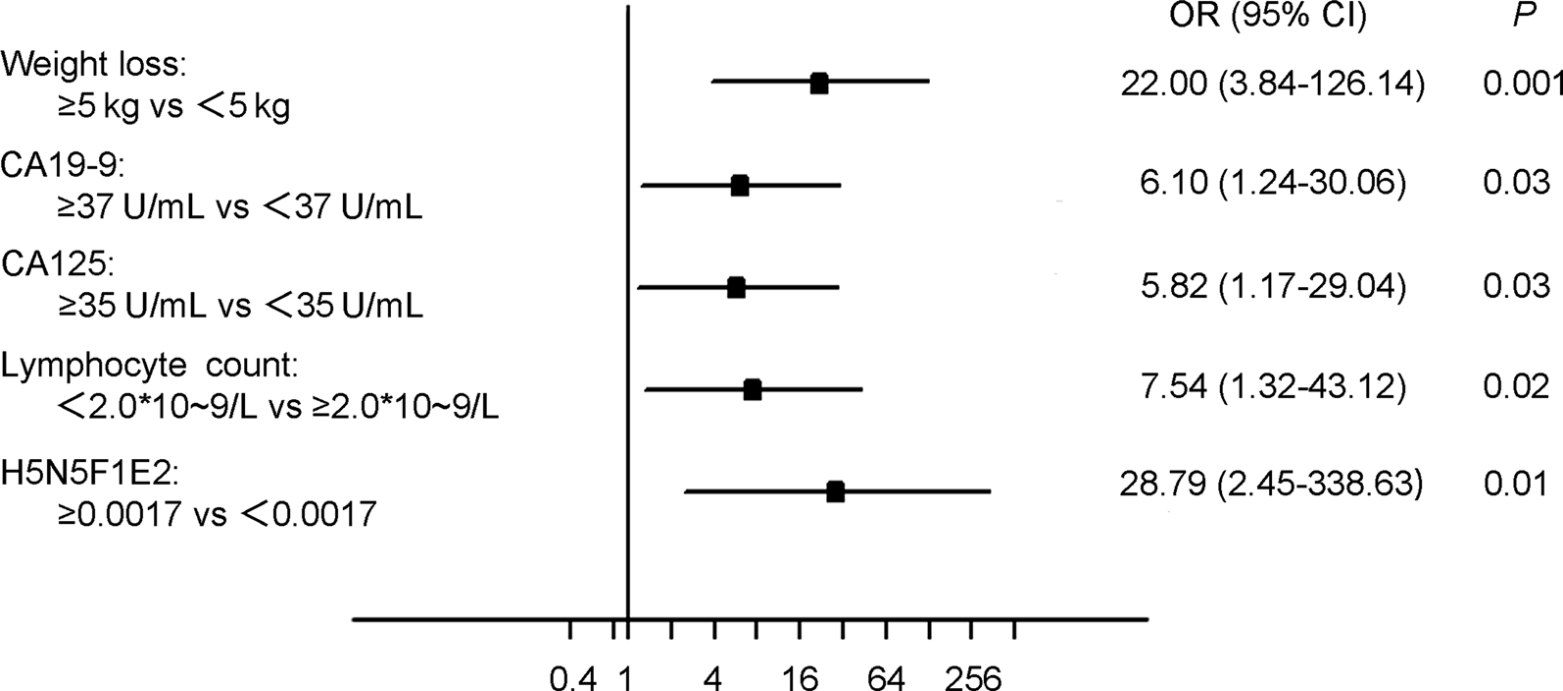
Forest Plot of multivariable logistic analysis identified independent risk factors for peritoneal metastasis in gastric cancer patients. CA19-9, CA125, CA724: carbohydrate antigen 19-9, 125, and 724; *H* hexose, *N* *N*-acetylhexosamine, *F* fucose, *L* α 2,3-sialic acid, *E* α 2,6-sialic acid

To identify the odds ratio (OR) and 95% confidence interval (CI) of the risk factors for peritoneal metastasis in gastric cancer patients, univariable and multivariable analysis were conducted using a logistic regression model. Weight loss ≥ 5 kg (excluding the reason for gastroduodenal obstruction), tumour size ≥ 3 cm, signet ring cell or mucinous adenocarcinoma histology type, poor differentiation, diffuse or mixed Lauren classification, increased carbohydrate antigens (CA19-9, CA125, CA724), decreased lymphocyte count, and haemoglobin, albumin, pre-albumin, and increased N-glycan levels were identified as risk factors for peritoneal metastasis (Table 1). All the potential risk factors with *P* < 0.05 identified from the univariate survival analysis were included in the multivariable logistic analysis. Weight loss ≥ 5 kg (OR: 22.00, 95% CI 3.84–126.14, *P* = 0.001), CA19-9 ≥ 37 U/mL (OR: 6.10, 95% CI 1.24–30.06, *P* = 0.03), CA125 ≥ 35 U/mL (OR: 5.82, 95% CI 1.17–29.04, *P* = 0.03), lymphocyte count < 2.0 * 10–9/L (OR: 7.54, 95% CI 1.32–43.12, *P* = 0.02), and H5N5F1E2 (m/z 2650.98) expression ≥ 0.0017 (OR: 29.79, 95% CI 2.45–338.63, *P* = 0.01) were identified as independent risk factors for peritoneal metastasis after the adjustment of covariates (Fig. 3).

**Table 1:** Univariate logistic analysis for peritoneal metastasis of gastric cancer

	Gastric cancer patients			*P*
	Total: n = 129 (%)	No PM: n = 86 (%)	PM: n = 43 (%)	
Age				
Mean(min–max)		29–84 (59.0)	32–81 (59.5)	0.32
≤ 60	65 (50)	46 (53)	19 (44)	
> 60	64 (50)	40 (47)	24 (56)	
Gender				0.24
Male	84 (65)	59 (69)	25 (58)	
Female	45 (35)	27 (31)	18 (42)	
Weight loss				0.001
No change	84 (65)	64 (74)	20 (47)	
< 5 kg	29 (22)	17 (20)	12 (27)	
≥ 5 kg	16 (12)	5 (6)	11 (26)	
Tumor location				0.008
Upper 1/3	20 (16)	13 (15)	7 (16)	
Middle 1/3	38 (29)	24 (28)	14 (33)	
Lower 1/3	57 (44)	45 (52)	12 (27)	
Mixed	13 (10)	4 (5)	9 (22)	
Others^a^	1 (1)	0 (0)	1 (2)	
Tumor size				0.001
Mean (min–max)		0.5–14 (3.5)	1–16 (5.9)	
< 3 cm	47 (36)	40 (47)	7 (16)	
≥ 3 cm	82 (64)	46 (53)	36 (84)	
Histology type				0.007
Adenocarcinoma	49 (38)	41 (48)	8 (19)	
Others	74 (57)	45 (52)	29 (67)	
Data absent	6 (5)	0 (0)	6 (14)	
Differentiation				0.004
High + moderate	18 (14)	18 (21)	0 (0)	
Poor	105 (81)	68 (79)	37 (86)	
Data absent	6 (5)	0 (0)	6 (14)	
Lauren classification				0.008
Intestinal	34 (26)	30 (35)	4 (9)	
Diffuse	41 (32)	23 (27)	18 (42)	
Mixed	46 (36)	29 (34)	17 (37)	
Data absent	8 (6)	4 (5)	4 (9)	
CEA				0.08
Mean (min–max)		0.2–55.9 (3.4)	0.4–372.8 (17.4)	
< 5 ng/mL	105 (81)	73 (85)	32 (74)	
≥ 5 ng/mL	22 (17)	11 (13)	11 (26)	
Data absent	2 (2)	2 (2)	0 (0)	
CA19-9				< 0.001
Mean (min–max)		0.6–2501 (41.9)	0.6–1000 (437.4)	
< 37 U/mL	105 (81)	80 (93)	25 (58)	
≥ 37 U/mL	21 (16)	6 (7)	15 (35)	
Data absent	3 (2)	0 ()	3 (7)	
CA125				< 0.001
Mean (min–max)		3.4–49.1 (13.0)	7.5–129.3 (35.2)	
< 35 U/mL	101 (78)	76 (88)	25 (58)	
≥ 35 U/mL	19 (15)	6 (7)	13 (30)	
Data absent	9 (7)	4 (5)	5 (12)	
CA724				0.002
Mean (min–max)		0.8–84.6 (5.9)	0.9–300 (34.9)	
< 10 U/mL	88 (68)	68 (79)	20 (47)	
≥ 10 U/mL	26 (20)	12 (14)	14 (33)	
Data absent	15 (12)	6 (7)	9 (20)	
AFP				0.16
Mean (min–max)		0.2–11.5 (2.4)	1–15.3 (3.8)	
< 20 ng/mL	119 (92)	80 (93)	39 (91)	
≥ 20 ng/mL	1 (1)	0 (0)	1 (2)	
Data absent	9 (7)	6 (7)	3 (7)	
Neutrophil count				0.36
Mean (min–max)		1.6–16.2 (3.8)	1.6–7.9 (3.9)	
< 2.5 * 10–9/L	27 (21)	20 (23)	7 (16)	
≥ 2.5 * 10–9/L	102 (79)	66 (77)	36 (84)	
Lymphocyte count				0.001
Mean (min–max)		1.1–3.7 (1.79)	0.6–2.7 (1.46)	
< 2.0 * 10–9/L	94 (73)	55 (64)	39 (91)	
≥ 2.0 * 10–9/L	35 (27)	31 (36)	4 (9)	
NLR				0.006
Mean (min–max)		0.7–16.2 (2.4)	0.9–11.3 (2.9)	
< 2.0	64 (50)	50 (58)	14 (33)	
≥ 2.0	65 (50)	36 (42)	29 (67)	
Haemoglobin				0.003
Mean (min–max)		52–160 (126.2)	74–159 (113.7)	
< 133 g/L	79 (61)	45 (52)	34 (79)	
≥ 133 g/L	50 (39)	41 (48)	9 (21)	
Platelet count				0.19
Mean (min–max)		96–419 (233.5)	96–505 (234.4)	
< 202 * 10–9/L	43 (33)	32 (37)	11 (26)	
≥ 202 * 10–9/L	86 (67)	54 (63)	32 (74)	
Albumin				0.009
Mean (min–max)		29–49 (39.6)	30–47 (38.9)	
< 37 g/L	38 (29)	19 (22)	19 (44)	
≥ 37 g/L	91 (71)	67 (78)	24 (56)	
Pre-albumin				< 0.001
Mean (min–max)		0.14–0.42 (0.25)	0.09–0.35 (0.21)	
< 0.21 g/L	37 (29)	15 (17)	22 (51)	
≥ 0.21 g/L	92 (71)	71 (83)	21 (49)	
H6N5L1E1: m/z 2620.93				< 0.001
Mean (min–max)		0.001–0.048 (0.006)	0.002–0.020 (0.007)	
< 0.0038	49 (38)	43 (50)	6 (14)	
≥ 0.0038	80 (62)	43 (50)	37 (86)	
H5N5F1E2: m/z 2650.98				< 0.001
Mean (min–max)		0.001–0.028 (0.003)	0.002–0.013 (0.004)	
< 0.0017	33 (26)	32 (37)	1 (2)	
≥ 0.0017	96 (74)	54 (63)	42 (98)	
H6N5E2: m/z 2666.96				< 0.001
Mean (min–max)		0.004–0.008 (0.003)	0.002–0.010 (0.004)	
< 0.0020	42 (33)	39 (45)	3 (7)	
≥ 0.0020	87 (67)	47 (55)	40 (93)	
H6N5L1E2: m/z 2940.08				< 0.001
Mean (min–max)		0.001–0.073 (0.010)	0.003–0.031 (0.013)	
< 0.0062	54 (42)	46 (53)	8 (19)	
≥ 0.0062	75 (58)	40 (47)	35 (81)	

*PM* peritoneal metastasis, *H* hexose, *N* *N*-acetylhexosamine, *F* fucose, *L* α 2,3-sialic acid, *E* α 2,6-sialic acid, *CA19*-*9, CA125, CA724* carbohydrate antigen 19-9, 125, and 724; NLR: neutrophil/lymphocyte ratio

*P* < 0.05 marked in bold font shows statistical significant

^a^Others include remnant stomach and anastomotic site

### Nomogram for predicting the risk of peritoneal metastasis

**Fig. 4:**
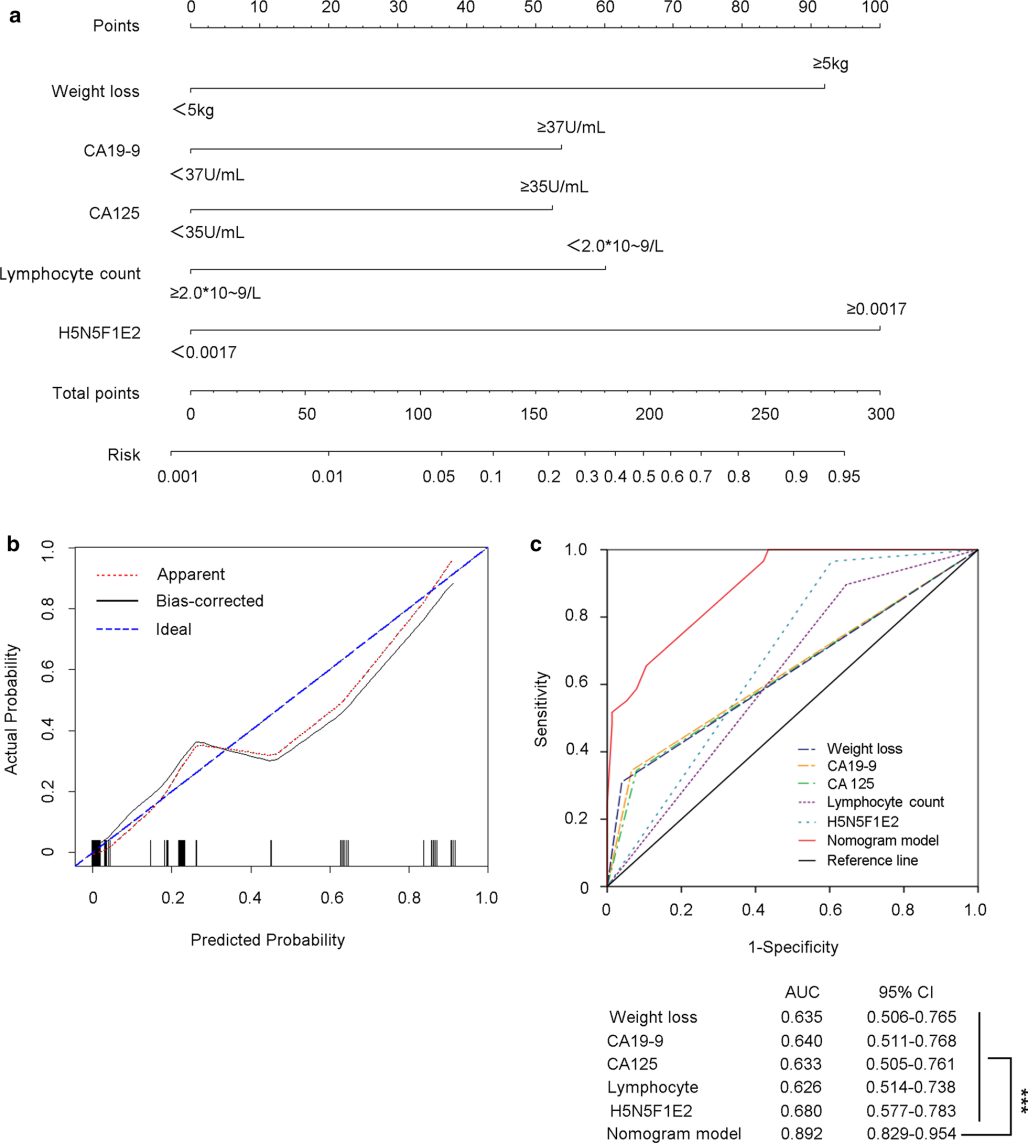
Establishment and validation of nomogram to predict the risk of peritoneal metastasis in gastric cancer patients. **a** A nomogram integrating with weight loss, CA19-9, CA-125, lymphocytes count number, and H5N5F1E2 level was created to predict risk of peritoneal metastasis. **b** Calibration curve for nomogram-predicted and actual probability of having peritoneal metastasis. **c** Sensitivity and specificity for predicting peritoneal metastasis of nomogram model and other single variables was compared by the area under the Receiver operating characteristic (ROC) curve (AUC) and tested by Delong. Delong. Clarke-Pearson test. CA19-9, CA125, CA724: carbohydrate antigen 19-9, 125, and 724; *H* hexose, *N* *N*-acetylhexosamine, *F* fucose, *L* α 2,3-sialic acid, *E* α 2,6-sialic acid. ***P < 0.001

To provide a prediction model for peritoneal metastasis in gastric cancer patients before surgery, a nomogram based on the results from the stepwise logistic regression model was created to provide a quantitative method for better prediction (Fig. 4a).

In the nomogram, each variable was represented by a different point, which was listed in Additional file 1: Table S4. The total number of points was calculated by adding the point of each variable, and a higher total number of points represented a higher risk of peritoneal metastasis.

### Validation of the prediction model

The prediction model of the nomogram was validated by measuring the calibration and AUC. The calibration was evaluated with a calibration curve, in which patients were grouped by predicted risk, which was then plotted as actual vs. predicted risk. As shown in Fig. 4b, the calibration plot demonstrated that the nomogram performed well compared with the ideal prediction model. The ROC curve showed that the nomogram (AUC: 0.892, 95% CI 0.829-0.954) displayed the best sensitivity and specificity for predicting peritoneal metastasis compared with any other single variable (*P* < 0.001) (Fig. 4c).

### Illustration for clinical practice

**Fig. 5:**
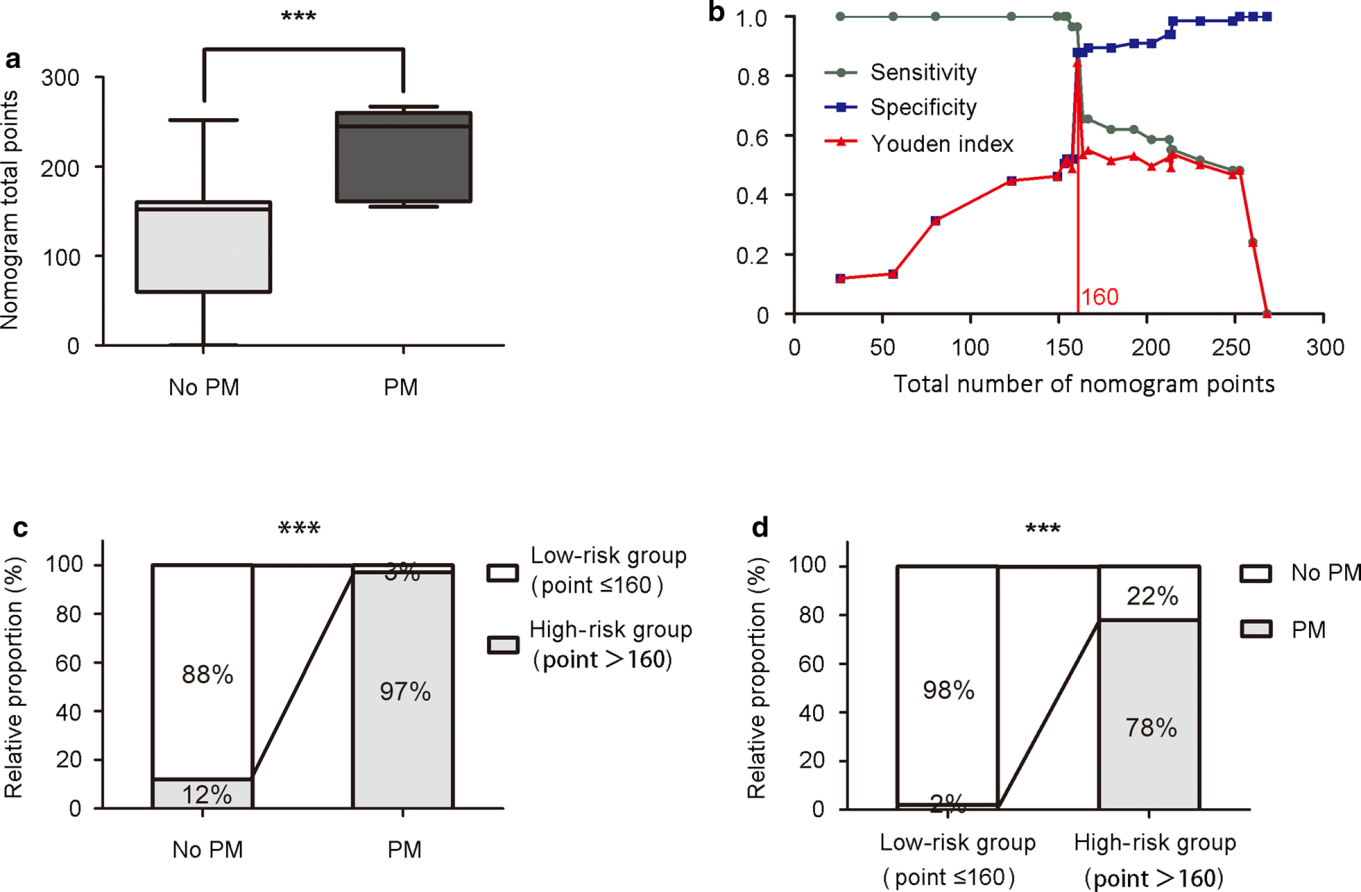
Clinical significance of nomogram to predict peritoneal metastasis. **a** Total number of nomogram points in non-peritoneal metastasis (Mean ± SEM: 118.6 ± 7.93) and peritoneal metastasis (Mean ± SEM: 216.4 ± 8.77) patients were demonstrated by box plot. The box plot shows the full range of variation (error bars: min and max) with the line representing median. **b** When 160 was set as the cut-off value determined by ROC analysis and Youden index, nomogram had the best sensitivity (0.97) and specificity (0.88). Youden index = Sensitivity + Specificity − 1. **c** Positive rate (97%), negative rate (88%), false positive rate (12%), and false negative rate (3%) of the nomogram stratified into low-risk group (total number of nomogram points ≤ 160) and high-risk group (total number of nomogram points > 160). **d** Proportion of patients with or without peritoneal metastasis was demonstrated in low-risk group and high-risk group. *PM* peritoneal metastasis; ***P < 0.001

The number of nomogram points was significantly higher in patients with peritoneal metastasis than in patients without distant metastasis (Fig. 5a). Optimal cut-off threshold values were determined at the point on the ROC curve at which the Youden’s index (sensitivity + specificity − 1) was maximal [8]. The result showed that when 160 was set as the cut-off value, the nomogram had the best discriminating power considering both sensitivity (0.97) and specificity (0.88) (Fig. 5b). Therefore, patients could be stratified into a low-risk group (total number of nomogram points ≤ 160) and a high-risk group (> 160). Only 12% of patients without PM were in the high-risk group (false-positive rate); however, the proportion increased to 97% in PM patients (positive rate) (Fig. 5c). Furthermore, the proportion of PM increased from 2% in the low-risk group to 78% in the high-risk group (Fig. 5d).

## Discussion

Peritoneal metastasis is regarded as the most prevalent incurable cause of gastric cancer. In our 728 consecutive gastric cancer patients, peritoneal metastasis was also identified as the most common mode of distant metastasis (47/65: 72%). In patients with peritoneal metastasis, although controversy regarding surgical application still remains, palliative chemotherapy is preferred [14]. From this point of view, peritoneal metastasis needs to be precisely diagnosed before surgery or at the beginning of surgery in order for surgeons to determine the most appropriate therapeutic approach and to avoid unnecessary extensive surgery [6].

However, in clinical practice, it is often difficult to make diagnosis of peritoneal metastasis by conventional imaging modalities, such as CT, PET-CT or MRI, due to their limitations in terms of detection sensitivity [5]. Consistent with previous studies showing that peritoneal metastasis is one of the limitations of CT or PET-CT for predicting the stage of gastric cancer preoperatively [15, 16], CT had a very low sensitivity for detecting peritoneal metastasis (25.7%), and PET-CT achieved a moderately higher sensitivity of 45.5% in our study, although both showed good performance in terms of diagnostic accuracy (100%). CT had an advantage in terms of diagnostic performance with lesions of 10 mm or more in diameter; however, the majority of peritoneal metastasis cases showed numerous miliary nodules with diffuse and random distribution in the uneven shape of the peritoneal cavity or omental burse, making it difficult for the entire cavity to be thoroughly inspected. The function of PET-CT depends on the cellular FDG (^18^F-fluoro-2-deoxyglucose) uptake, which is predominantly related to glucose transporter 1 (GLUT1) expression [17]. GLUT1 is usually overexpressed in malignant tissues, leading to the intracellular accumulation of FDG, which can then be visualized by PET-CT [18]. However, GLUT1 expression varies greatly in different gastric cancer histologies. Signet ring cell carcinoma, mucinous adenocarcinoma, or poorly differentiated adenocarcinoma, which are the most common histological types of peritoneal metastasis, show very low positive values for GLUT1 expression [19]. Consequently, new method are urgently needed to improve the preoperative diagnosis of peritoneal metastasis.

Clinicopathologic characteristics and serum biomarkers were first explored to distinguish peritoneal metastasis. A large tumour size, diffuse types, poor differentiation, or mucinous adenocarcinoma histopathological type have been reported to be positively associated with peritoneal metastasis [8], which is consistent with our results. The most frequently used tumour biomarkers, such as CA19-9 [20], CA12-5, and CA-724 [2], have also been identified to provide additional information for the diagnosis of peritoneal metastasis. In our study, we found a significant increased risk of peritoneal metastasis in patients with elevated serum biomarkers. CA12-5 has been reported to be expressed by the epithelium of the mesothelium lining body cavities (pleural, peritoneal, and pelvic cavities) [21]. As a result, in the case of peritoneal metastasis, the overexpression of CA12-5 is caused not only by tumour burden but also, more importantly, by peritoneal or endometrial inflammation induced by tumour dissemination to the mesothelium lining body cavities [22, 23]. CA724 has been found to be elevated in adenocarcinomas, including gastric cancer, especially in the mucinous sub-type [24]. Peritoneal metastasis in gastric cancer mainly occurs in histological types of mucinous adenocarcinoma or signet ring cell adenocarcinoma, both of which are rich in mucus. This may explain why patients with peritoneal metastasis usually have elevated CA724 levels.

Recently, some markers reflecting the systemic inflammatory response or immune responses of the host in patients with cancer have been indicated to be associated with cancer metastasis [25, 26]. A correlation between an increase in serum CRP level or a decrease in serum albumin levels with the presence of peritoneal metastasis has been discovered [7, 8]. Increased neutrophil counts, decreased lymphocyte counts, and the neutrophil/lymphocyte ratio (NLR) have also been demonstrated to facilitate the diagnosis of peritoneal metastasis [7]. Data from our study revealed that a decreased lymphocyte count, an increased NLR, a decreased haemoglobin level, and a decreased albumin or pre-albumin levels in preoperative serum were all associated with peritoneal metastasis. However, only lymphocyte count remained significant in the multivariable logistic analysis, which was consistent with previous results showing that using lymphocyte count alone is superior to NLR for the diagnosis of peritoneal metastasis [8]. Lymphocytes have been reported to reflect the defensive activity of the host against tumours [27]; thus, a reduced number of lymphocytes may facilitate tumour progression.

In addition to these clinicopathologic characteristics and laboratory parameters reported previously, we first identified PM specifically related serum glycans by MALDI-TOF MS and glycomic profile analysis. Glycosylation is a kind of posttranslational modification in the majority of proteins to modulate and control their biological roles. Abnormal glycosylation is associated with tumour progression and metastasis in a variety of cancers [28–31]. It is worth noting that the alteration of *N*-glycans is implicated in the modulation of cell-ECM (extracellular matrix) associations as well as in cell–cell adhesion and migration, which are closely related to the metastasis potential of tumours [32]. The role of glycosylation modifications has also been associated with peritoneal metastasis. For example, mesothelin-MUC16 binding that facilitates peritoneal metastasis in ovarian cancers has been shown to be dependent on N-glycan [33]. As a result, tumour metastasis-associated aberrations in glycan structures may provide a compelling rationale for the discovery of new biomarkers. To date, glycan alterations in gastric cancer have only been reported in very few studies, and most of the studies identified limited *N*-glycans for providing comprehensive glycomic analysis due to the insufficient sensitivity of the methods [34, 35]. In this study, we applied MALDI-TOF MS, a highly developed and powerful technology, for the qualitative and quantitative analysis of glycans [10, 36]. Compared with non-metastatic serum samples, 22 *N*-glycans showed significant difference in PM samples. Among these N-glycans, the abundances of 9 N-glycans (H5N4FIL1E1, H6N6E1, H6N5L2, H6N5L1E1, H5N5F1E2, H6N5E2, H6N5L2E1, H6N5L1E2, H6N5E3) containing sialylation were remarkably higher in PM samples. Sialic acids were reported to play important roles in cell–cell interaction, recognition and immunological response [37], as well as specific biomarkers for tumor progression [38]. Besides, two of 22 *N*-glycans were high mannose glycans (H6N2, H8N2), which were reported to be abundantly expressed in metastatic cholangiocarcinoma, and promote metastasis of cholangiocarcinoma by enhancing the ability to translocate, invade surrounding basement membrane matrix, and migrate [39]. We also found 2 of 22 *N*-glycans were bisecting GlcNAc (H5N5F2, H5N5E1AC2), which showed significantly decreased levels in PM. N-glycans with bisecting GlcNAc have been identified to be valuable for detecting the early peritoneal metastasis in ovarian cancer [40].

Though these PM-associated variables were identified, the diagnostic accuracy of each single parameter was underpowered. Some variables, such as lymphocyte count and H5N5F1E2 expression level, achieved good sensitivity but had low specificity. However, others showed better specificity than sensitivity, such as weight loss, CA19-9 and CA-125 levels. Thus, a nomogram was introduced here to build a statistical model for evaluating the risk of peritoneal metastasis. Many investigations have shown that the nomogram is a visualized graphical statistical model for the individualized assessment of the quantified risk of clinical events by a variety of factors [41, 42]. In our study, as illustrated in Fig. 4, a nomogram was established to predict the risk of peritoneal metastasis to facilitate clinical decision-making. If a patient lost less than 5 kg of body weight, and had elevated CA199 and CA-125 levels but did not have an abnormal decreased lymphocyte count and increased H5N5F1E2 expression (total points = 0 + 54 + 53 + 0 + 0 = 107), then the risk of peritoneal metastasis was less than 5%. If the patients had increased H5N5F1E2 expression, then the points reached 207 (107 + 100 = 207), with a corresponding risk of approximately 50%. We further stratified patients into a low-risk group (total number of nomogram points ≤ 160) and a high-risk group (total number of nomogram points > 160) according to the ROC analysis. Hence, surgeons can make treatment plans with the visual assistance of the nomogram preoperatively.

## Conclusions

Patients with gastric cancer showed an increased risk of developing peritoneal metastasis with more than 5 kg of body weight loss, elevated CA19-9 and CA-125 levels, decreased lymphocyte counts, and increased H5N5F1E2 expression. By incorporating these five variables, a nomogram predicting peritoneal metastasis was developed. Then, the incidence of peritoneal metastasis in an individual gastric cancer patient could be estimated. However, further internal and external validation of the application of the nomogram model is required.

## Supplementary information

**Additional file 1: Figure S1.** Representative MALDI-TOF spectra of serum N-glycomics profile in gastric cancer. **Table S1.** Consecutive GC patients treated in Zhongshan Hospital from April 2015 to November 2015. **Table S2.** Compositions detected by positive reflectron mode MALDI-TOF-MS after ethyl esterification. **Table S3.** List of the 22 serum N-glycans that were evaluated to be significantly different between non-metastatic GC and PMGC. **Table S4.** Nomogram point of each variable.

## Data Availability

All the data, analytic methods, and study materials supporting the findings of this study are available from the corresponding authors upon reasonable request.

## Abbreviations

GC: Gastric cancer
PM: Peritoneal metastasis
MALDI-TOF MS: Matrix-assisted laser desorption/ionization time-of-flight mass spectrometry
CA: Carbohydrate antigens
CRP: C-reactive protein
NLR: Neutrophil to lymphocyte ratio
ROC: Receiver operating characteristic curve
AUC: Area under ROC curve
H: Hexose
N: *N*-Acetylhexosamine
F: Fucose
L: α 2,3-sialic acid
E: α 2,6-Sialic acid
OR: Odds ratio
CI: Confidence interval
FDG: F-Fluoro-2-deoxyglucose
GLUT1: Glucose transporter 1
