# Supplementary material for: A nomogram based on glycomic biomarkers in serum and clinicopathological characteristics for evaluating the risk of peritoneal metastasis in gastric cancer
Source: Clin Proteomics. 2020 Sep 19;17:34. doi: 10.1186/s12014-020-09297-4 (PMC7501696; doi:10.1186/s12014-020-09297-4)
Supplement: Supplementary file 1 — Additional file 1: Figure S1. Representative MALDI-TOF spectra of serum N-glycomics profile in gastric cancer. Table S1. Consecutive GC patients treated in Zhongshan Hospital from April 2015 to November 2015. Table S2. Compositions detected by positive reflectron mode MALDI-TOF-MS after ethyl esterification. Table S3. List of the 22 serum N-glycans that were evaluated to be significantly different between non-metastatic GC and PMGC. Table S4. Nomogram point of each variable. [file 12014_2020_9297_MOESM1_ESM.docx]

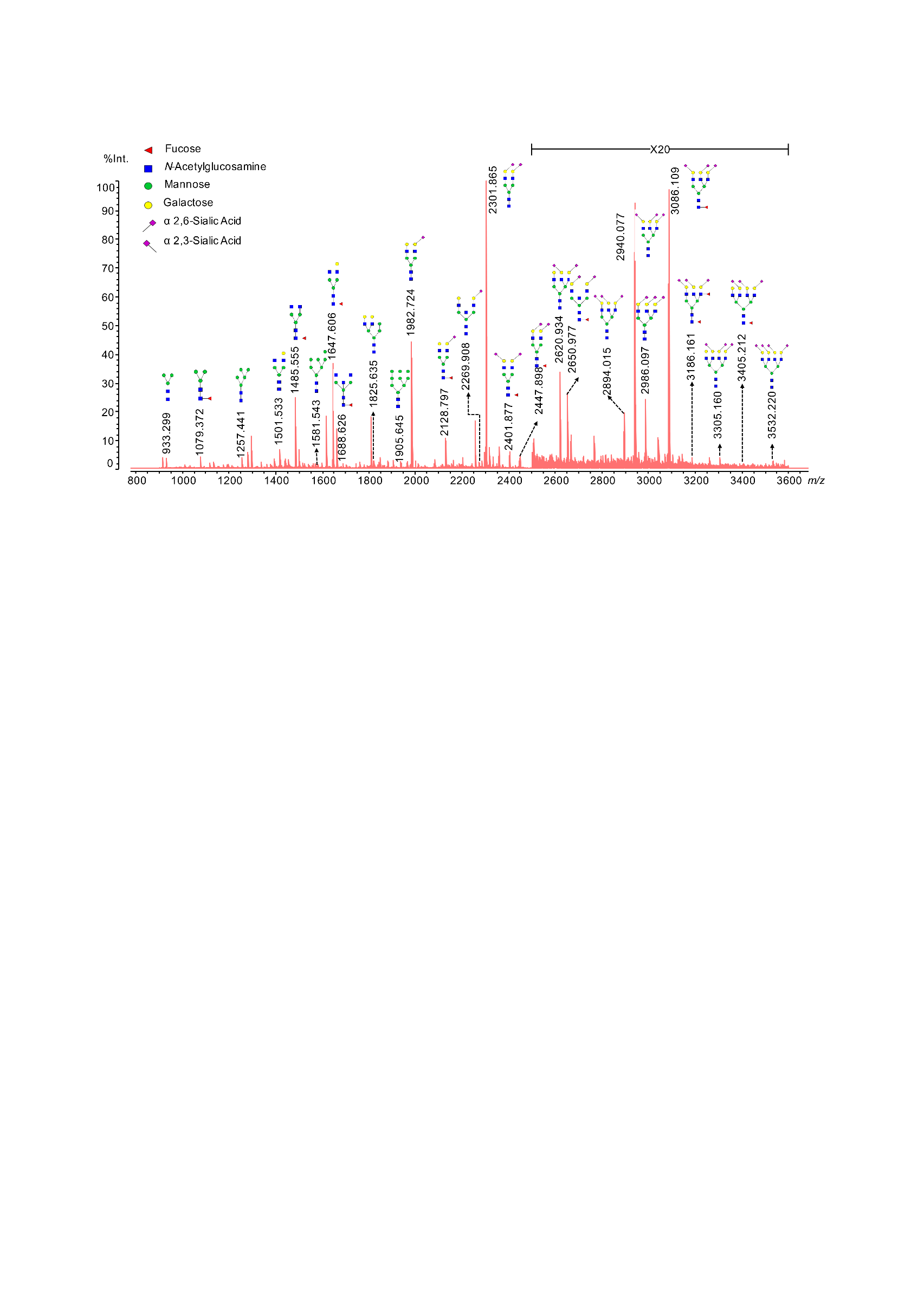
**Additional Figure**

**Figure S1. Representative MALDI-TOF spectra of serum N-glycomics profile in gastric cancer.** Representative peaks have been structurally annotated with putative structures in terms of N-acetylglucosamine, mannose, galactose, fucose, and Sialic acids. No linkage information is obtained; therefore, putative linkages are displayed.

**Additional Table**

| **Table S1.** Consecutive GC patients treated in Zhongshan Hospital from April 2015 to November 2015 | |
| --- | --- |
|  | **Number of patients (%)** |
| **All patients** | 728 (100) |
| **Stage I** | 220 (30.2) |
| **Stage II** | 142 (19.5) |
| **Stage III** | 301 (41.3) |
| **Stage IV** | 65 (9.0) |
| Peritoneal metastasis alone | 42 (64.6) |
| Liver metastasis alone | 12 (18.5) |
| Adnexa uteri alone | 3 (4.6) |
| Left supraclavicular lymph nodes alone | 2 (3.1) |
| Bone metastasis alone | 1 (1.5) |
| Multiple metastasis | 5 (7.7) |
| Peritoneal & Liver | 3 |
| Peritoneal & Left supraclavicular lymph nodes | 1 |
| Multiple sites (including peritoneal metastasis) | 1 |
| **Note.** GC: gastric cancer. |  |

| **Table S2.** Compositions detected by positive reflectron mode MALDI-TOF-MS after ethyl esterification | | | | | | | | | | | | |
| --- | --- | --- | --- | --- | --- | --- | --- | --- | --- | --- | --- | --- |
| **Observed peaks m/z** | **Input composition** | | | | | **Modifiers** | | | **Calculated** | | **Deviation (observed vs. calculated)** | |
|  | H | N | F | L | E | H2O | Na+ | Ac | Composition | Mass | error (Da) | error (ppm) |
|  | 162.05 | 203.08 | 146.06 | 273.08 | 319.13 | 18.01 | 22.99 | 42.01 |  |  |  |  |
| 933.3 | 3 | 2 | 0 | 0 | 0 | 1 | 1 | 0 | H3N2 | 933.32 | -0.02 | -18.29 |
| 1079.4 | 3 | 2 | 1 | 0 | 0 | 1 | 1 | 0 | H3N2F1 | 1079.37 | 0 | -2.29 |
| 1095.4 | 4 | 2 | 0 | 0 | 0 | 1 | 1 | 0 | H4N2 | 1095.37 | -0.02 | -17.23 |
| 1136.4 | 3 | 3 | 0 | 0 | 0 | 1 | 1 | 0 | H3N3 | 1136.4 | 0.01 | 12.56 |
| 1257.4 | 5 | 2 | 0 | 0 | 0 | 1 | 1 | 0 | H5N2 | 1257.42 | 0.02 | 14.53 |
| 1282.4 | 3 | 3 | 1 | 0 | 0 | 1 | 1 | 0 | H3N3F1 | 1282.45 | -0.01 | -8.04 |
| 1298.5 | 4 | 3 | 0 | 0 | 0 | 1 | 1 | 0 | H4N3 | 1298.45 | 0.01 | 9.51 |
| 1339.5 | 3 | 4 | 0 | 0 | 0 | 1 | 1 | 0 | H3N4 | 1339.48 | 0.01 | 4.8 |
| 1419.5 | 6 | 2 | 0 | 0 | 0 | 1 | 1 | 0 | H6N2 | 1419.48 | 0.02 | 11.6 |
| 1444.5 | 4 | 3 | 1 | 0 | 0 | 1 | 1 | 0 | H4N3F1 | 1444.51 | -0.01 | -8.38 |
| 1455.5 | 3 | 3 | 0 | 0 | 1 | 1 | 1 | 0 | H3N3E1 | 1455.52 | -0.01 | -7.64 |
| 1460.5 | 5 | 3 | 0 | 0 | 0 | 1 | 1 | 0 | H5N3 | 1460.5 | 0.01 | 7.23 |
| 1485.6 | 3 | 4 | 1 | 0 | 0 | 1 | 1 | 0 | H3N4F1 | 1485.53 | 0.02 | 14.16 |
| 1501.5 | 4 | 4 | 0 | 0 | 0 | 1 | 1 | 0 | H4N4 | 1501.53 | 0 | 3 |
| 1542.5 | 3 | 5 | 0 | 0 | 0 | 1 | 1 | 0 | H3N5 | 1542.56 | -0.04 | -26.24 |
| 1581.5 | 7 | 2 | 0 | 0 | 0 | 1 | 1 | 0 | H7N2 | 1581.53 | 0.01 | 9.2 |
| 1617.6 | 4 | 3 | 0 | 0 | 1 | 1 | 1 | 0 | H4N3E1 | 1617.58 | 0.03 | 16.08 |
| 1622.6 | 6 | 3 | 0 | 0 | 0 | 1 | 1 | 0 | H6N3 | 1622.55 | 0.01 | 5.32 |
| 1647.6 | 4 | 4 | 1 | 0 | 0 | 1 | 1 | 0 | H4N4F1 | 1647.59 | 0.02 | 11.6 |
| 1663.6 | 5 | 4 | 0 | 0 | 0 | 1 | 1 | 0 | H5N4 | 1663.58 | 0 | 1.63 |
| 1688.6 | 3 | 5 | 1 | 0 | 0 | 1 | 1 | 0 | H3N5F1 | 1688.61 | 0.01 | 7.81 |
| 1704.6 | 4 | 5 | 0 | 0 | 0 | 1 | 1 | 0 | H4N5 | 1704.61 | 0 | -1.88 |
| 1743.6 | 8 | 2 | 0 | 0 | 0 | 1 | 1 | 0 | H8N2 | 1743.58 | 0.01 | 7.32 |
| 1763.6 | 4 | 3 | 1 | 0 | 1 | 1 | 1 | 0 | H4N3F1E1 | 1763.63 | 0 | 0.88 |
| 1779.7 | 5 | 3 | 0 | 0 | 1 | 1 | 1 | 0 | H5N3E1 | 1779.63 | 0.02 | 13.61 |
| 1809.7 | 5 | 4 | 1 | 0 | 0 | 1 | 1 | 0 | H5N4F1 | 1809.64 | 0.02 | 9.57 |
| 1820.7 | 4 | 4 | 0 | 0 | 1 | 1 | 1 | 0 | H4N4E1 | 1820.66 | 0.02 | 10.05 |
| 1825.6 | 6 | 4 | 0 | 0 | 0 | 1 | 1 | 0 | H6N4 | 1825.63 | 0 | 0.5 |
| 1850.7 | 4 | 5 | 1 | 0 | 0 | 1 | 1 | 0 | H4N5F1 | 1850.67 | 0.01 | 6.09 |
| 1866.7 | 5 | 5 | 0 | 0 | 0 | 1 | 1 | 0 | H5N5 | 1866.66 | -0.01 | -2.75 |
| 1905.6 | 9 | 2 | 0 | 0 | 0 | 1 | 1 | 0 | H9N2 | 1905.63 | 0.01 | 5.69 |
| 1936.7 | 5 | 4 | 0 | 1 | 0 | 1 | 1 | 0 | H5N4L1 | 1936.67 | 0.04 | 19.11 |
| 1941.7 | 6 | 3 | 0 | 0 | 1 | 1 | 1 | 0 | H6N3E1 | 1941.68 | 0.02 | 11.48 |
| 1966.8 | 4 | 4 | 1 | 0 | 1 | 1 | 1 | 0 | H4N4F1E1 | 1966.71 | 0.03 | 16.67 |
| 1982.7 | 5 | 4 | 0 | 0 | 1 | 1 | 1 | 0 | H5N4E1 | 1982.71 | 0.02 | 8.26 |
| 2012.7 | 5 | 5 | 1 | 0 | 0 | 1 | 1 | 0 | H5N5F1 | 2012.72 | 0.01 | 4.71 |
| 2023.8 | 4 | 5 | 0 | 0 | 1 | 1 | 1 | 0 | H4N5E1 | 2023.73 | 0.05 | 24.47 |
| 2067.7 | 10 | 2 | 0 | 0 | 0 | 1 | 1 | 0 | H10N2 | 2067.69 | 0.01 | 4.37 |
| 2082.8 | 5 | 4 | 1 | 1 | 0 | 1 | 1 | 0 | H5N4F1L1 | 2082.72 | 0.05 | 24.72 |
| 2128.8 | 5 | 4 | 1 | 0 | 1 | 1 | 1 | 0 | H5N4F1E1 | 2128.77 | 0.03 | 14.5 |
| 2158.8 | 5 | 5 | 2 | 0 | 0 | 1 | 1 | 0 | H5N5F2 | 2158.78 | -0.02 | -7 |
| 2169.8 | 4 | 5 | 1 | 0 | 1 | 1 | 1 | 0 | H4N5F1E1 | 2169.79 | 0.03 | 11.55 |
| 2185.8 | 5 | 5 | 0 | 0 | 1 | 1 | 1 | 0 | H5N5E1 | 2185.79 | 0.01 | 3.96 |
| 2209.8 | 5 | 4 | 0 | 2 | 0 | 1 | 1 | 0 | H5N4L2 | 2209.75 | 0.03 | 14.64 |
| 2227.8 | 5 | 5 | 0 | 0 | 1 | 1 | 1 | 1 | H5N5E1Ac1 | 2227.8 | 0 | -2.06 |
| 2231.8 | 6 | 6 | 0 | 0 | 0 | 1 | 1 | 0 | H6N6 | 2231.79 | -0.01 | -6.56 |
| 2255.8 | 5 | 4 | 0 | 1 | 1 | 1 | 1 | 0 | H5N4L1E1 | 2255.79 | 0.01 | 5.2 |
| 2269.9 | 5 | 5 | 0 | 0 | 1 | 1 | 1 | 2 | H5N5E1Ac2 | 2269.81 | 0.1 | 43.77 |
| 2301.9 | 5 | 4 | 0 | 0 | 2 | 1 | 1 | 0 | H5N4E2 | 2301.83 | 0.03 | 13.1 |
| 2331.9 | 5 | 5 | 1 | 0 | 1 | 1 | 1 | 0 | H5N5F1E1 | 2331.85 | 0.02 | 9.98 |
| 2347.9 | 6 | 5 | 0 | 0 | 1 | 1 | 1 | 0 | H6N5E1 | 2347.84 | 0.01 | 2.92 |
| 2355.8 | 5 | 4 | 1 | 2 | 0 | 1 | 1 | 0 | H5N4F1L2 | 2355.81 | 0.01 | 3.25 |
| 2372.9 | 4 | 6 | 1 | 0 | 1 | 1 | 1 | 0 | H4N6F1E1 | 2372.87 | 0.02 | 7.26 |
| 2377.9 | 6 | 6 | 1 | 0 | 0 | 1 | 1 | 0 | H6N6F1 | 2377.85 | 0 | -0.12 |
| 2401.9 | 5 | 4 | 1 | 1 | 1 | 1 | 1 | 0 | H5N4F1L1E1 | 2401.85 | 0.03 | 10.86 |
| 2429.9 | 4 | 7 | 0 | 0 | 1 | 1 | 1 | 0 | H4N7E1 | 2429.89 | 0.03 | 13.93 |
| 2447.9 | 5 | 4 | 1 | 0 | 2 | 1 | 1 | 0 | H5N4F1E2 | 2447.89 | 0.01 | 2.23 |
| 2477.9 | 5 | 5 | 2 | 0 | 1 | 1 | 1 | 0 | H5N5F2E1 | 2477.9 | 0 | -0.58 |
| 2493.9 | 6 | 5 | 1 | 0 | 1 | 1 | 1 | 0 | H6N5F1E1 | 2493.9 | 0.02 | 8.51 |
| 2504.9 | 5 | 5 | 0 | 0 | 2 | 1 | 1 | 0 | H5N5E2 | 2504.91 | 0.02 | 8.91 |
| 2547.9 | 5 | 4 | 2 | 1 | 1 | 1 | 1 | 0 | H5N4F2L1E1 | 2547.91 | 0.01 | 3.42 |
| 2550.9 | 6 | 6 | 0 | 0 | 1 | 1 | 1 | 0 | H6N6E1 | 2550.92 | -0.03 | -12.92 |
| 2574.9 | 6 | 5 | 0 | 2 | 0 | 1 | 1 | 0 | H6N5L2 | 2574.88 | 0.03 | 11.57 |
| 2605 | 5 | 5 | 1 | 1 | 1 | 1 | 1 | 0 | H5N5F1L1E1 | 2604.93 | 0.03 | 9.82 |
| 2620.9 | 6 | 5 | 0 | 1 | 1 | 1 | 1 | 0 | H6N5L1E1 | 2620.93 | 0.01 | 3.5 |
| 2651 | 5 | 5 | 1 | 0 | 2 | 1 | 1 | 0 | H5N5F1E2 | 2650.97 | 0.01 | 1.96 |
| 2667 | 6 | 5 | 0 | 0 | 2 | 1 | 1 | 0 | H6N5E2 | 2666.97 | -0.01 | -4.2 |
| 2721 | 6 | 5 | 1 | 2 | 0 | 1 | 1 | 0 | H6N5F1L2 | 2720.94 | 0.01 | 2.05 |
| 2767 | 6 | 5 | 1 | 1 | 1 | 1 | 1 | 0 | H6N5F1L1E1 | 2766.98 | 0.02 | 8.77 |
| 2813 | 6 | 5 | 1 | 0 | 2 | 1 | 1 | 0 | H6N5F1E2 | 2813.02 | 0 | 1.29 |
| 2894 | 6 | 5 | 0 | 2 | 1 | 1 | 1 | 0 | H6N5L2E1 | 2894.01 | 0.01 | 1.77 |
| 2940.1 | 6 | 5 | 0 | 1 | 2 | 1 | 1 | 0 | H6N5L1E2 | 2940.05 | 0.02 | 8.1 |
| 2986.1 | 6 | 5 | 0 | 0 | 3 | 1 | 1 | 0 | H6N5E3 | 2986.09 | 0 | 1.07 |
| 3040.1 | 6 | 5 | 1 | 2 | 1 | 1 | 1 | 0 | H6N5F1L2E1 | 3040.07 | 0.02 | 6.65 |
| 3086.1 | 6 | 5 | 1 | 1 | 2 | 1 | 1 | 0 | H6N5F1L1E2 | 3086.11 | 0 | -0.13 |
| 3132.1 | 6 | 5 | 1 | 0 | 3 | 1 | 1 | 0 | H6N5F1E3 | 3132.15 | -0.02 | -6.64 |
| 3186.2 | 6 | 5 | 2 | 2 | 1 | 1 | 1 | 0 | H6N5F2L2E1 | 3186.13 | 0.04 | 11 |
| 3259.1 | 7 | 6 | 0 | 2 | 1 | 1 | 1 | 0 | H7N6L2E1 | 3259.14 | 0 | -1.01 |
| 3305.2 | 7 | 6 | 0 | 1 | 2 | 1 | 1 | 0 | H7N6L1E2 | 3305.18 | -0.02 | -7.24 |
| 3405.2 | 7 | 6 | 1 | 2 | 1 | 1 | 1 | 0 | H7N6F1L2E1 | 3405.2 | 0.01 | 3.39 |
| 3532.2 | 7 | 6 | 0 | 3 | 1 | 1 | 1 | 0 | H7N6L3E1 | 3532.23 | -0.01 | -2.08 |
| **Note.** H=hexose; N=N-acetylhexosamine; F=fucose; L=α 2,3-sialic acid; E=α 2,6-sialic acid. | | | | | | | | | | | | |

| **Table S3.** List of the 22 serum N-glycans that were evaluated to be significantly different between non-metastatic GC and PMGC | | | |
| --- | --- | --- | --- |
| **m/z** | **Composition** | ***P* value** | **AUC** |
| 1079.3724 | H3N2F1 | 0.0065 | 0.6347 |
| 1136.4106 | H3N3 | 0.0169 | 0.6074 |
| 1298.4615 | H4N3 | 0.0259 | 0.6025 |
| 1419.4919 | H6N2 | 0.0067 | 0.6531 |
| 1455.5119 | H3N3E1 | 0.0002 | 0.6750 |
| 1663.5841 | H5N4 | 0.0020 | 0.6814 |
| 1743.5938 | H8N2 | 0.0071 | 0.6206 |
| 1936.7032 | H5N4L1 | 0.0112 | 0.6360 |
| 2023.7841 | H4N5E1 | 0.0046 | 0.6398 |
| 2158.7614 | H5N5F2 | 0.0018 | 0.6487 |
| 2209.7834 | H5N4L2 | 0.0109 | 0.6168 |
| 2231.7783 | H6N6 | 0.0008 | 0.6682 |
| 2269.9079 | H5N5E1AC2 | 0.0024 | 0.6758 |
| 2401.8769 | H5N4FIL1E1 | 0.0021 | 0.6833 |
| 2550.8979 | H6N6E1 | 0.0337 | 0.6512 |
| 2574.9130 | H6N5L2 | 0.0026 | 0.6725 |
| 2620.9343 | H6N5L1E1 | < 0.0001 | 0.7355 |
| 2650.9772 | H5N5F1E2 | 0.0002 | 0.7226 |
| 2666.9558 | H6N5E2 | 0.0001 | 0.7115 |
| 2894.0151 | H6N5L2E1 | 0.0117 | 0.6812 |
| 2940.0756 | H6N5L1E2 | < 0.0001 | 0.7255 |
| 2986.0969 | H6N5E3 | 0.0105 | 0.6512 |

**Note.** GC: gastric cancer; PMGC: peritoneal metastasis gastric carcinoma; AUC: area under ROC curve. H: hexose; N: N-acetylhexosamine; F: fucose; L: α 2,3-sialic acid; E: α 2,6-sialic acid.

| **Table S4.** Nomogram point of each variable | |
| --- | --- |
| **Variables** | **Point** |
|  |  |
| **Weight loss** |  |
| <5kg | 0 |
| ≥5kg | 92 |
| **CA19-9** |  |
| <37U/mL | 0 |
| ≥37U/mL | 54 |
| **CA125** |  |
| <35U/mL | 0 |
| ≥35U/mL | 52 |
| **Lymphocyte count** |  |
| ≥2*10~9/L | 0 |
| <2*10~9/L | 60 |
| **H5N5F1E2: m/z 2650.98** |  |
| <0.0017 | 0 |
| ≥0.0017 | 100 |

**Note.** CA19-9, CA125: carbohydrate antigen 19-9,

and 125. H: hexose; N: N-acetylhexosamine;

F: fucose; L: α 2,3-sialic acid; E: α 2,6-sialic acid.
